# University of Zagreb Medical School Repository: promoting institutional visibility

**DOI:** 10.3325/cmj.2014.55.89

**Published:** 2014-04

**Authors:** Helena Markulin, Marijan Šember

**Affiliations:** Central Medical Library, University of Zagreb School of Medicine, Zagreb, Croatia

Scholarly journals are the main publication channel of many scientific disciplines. The advent of electronic journals has changed the publishing process and distribution of scientific information, creating new ways of journal access and use. At the same time, commercial publishers significantly increased the cost of access to scientific information, leading to dissatisfaction on the part of authors, academic institutions, and government funding bodies (1-3). The concept of research access crisis or journal pricing crisis refers to the existing dissatisfaction with journals publishing and distribution system. The academic institutions with their reduced budgets have become unable to keep pace with increases in journal publishing costs. Scientists and their institutions remain deprived even of the information they created themselves and freely ceded to the publishers (4). Suber defined the sources of their discontent as follows: *“*the… problem is that we donate time, labor, and public money to create new knowledge and then hand control over the results to businesses that believe, correctly or incorrectly, that their revenue and survival depend on limiting access to that knowledge*.”* (5).

A solution to this crisis was offered by open access (OA), which enables researchers to make their results freely available to worldwide research community. The main principles of the OA movement were defined by three international documents: the Budapest Open Access Initiative (February 2002) (6), the Bethesda Statement on Open Access Publishing (June 2003) (7), and the Berlin Declaration on Open Access (October 2003) (8). All three documents support the principle of free or minimally restricted access to peer-review research literature ceded by the authors to the publishers without any compensation. Such literature should be in digital format, online accessible, free of charge, and free of most copyright and licensing restrictions (4). There are many ways to deliver OA, such as personal websites, various Web 2.0 tools (wikis, blogs, social networks, etc), but two ways are dominant: “green” and “gold” OA. “Gold” OA is a type of OA where authors publish their manuscripts in open-access journals, while in “green” OA they archive them in a repository, which may be discipline-specific or institutional (9,10). Many of these manuscripts are already published, so the authors must comply with all the provisions of the publisher's Copyright Transfer Agreement. Authors can archive author’s manuscript prior to peer-review (the so-called pre-print), final peer-reviewed draft (eg, author’s post-print), or published versions of the manuscript. Although both OA roads, “green” and “gold,” allow free access to end-users, publishing costs still exist and are often met by authors themselves or by institutional or research funds (10,11).

## Open access movement in Croatia

The OA movement in Croatia has been embraced and supported by librarians, scientists, publishers, and government bodies. In 2004, Croatian Information and Documentation Society set up a working group to promote OA movement and launched a project called OASI – Open Access to Scientific Information. A number of Croatian scientific journals immediately accepted the initiative and started to publish OA articles on their websites. The online platform for local journals, named Hrčak, turned out to be one of the most successful national projects, comprising more than 350 fully or partially OA journals in 2014 (12). In 2010, The University of Zagreb adopted an OA statement requiring that all PhD candidates make their theses fully OA available on the university website (13). The Law on Science and Higher Education from 2013 mandates all universities and colleges to deposit a copy of all theses to the repository of the National and University Library (14). The legislative framework for the mandatory storage of other types of publications does not exist yet, though a document of the Croatian Government from 2005 states that “.... Scientific and technological system that is financed from public funds must be open to the public ... The results of research and development funded by public funds should be made available to the public in the form of open publications and open access databases ... ” (4). In October 2012, a group of Croatian scientists, librarians, and other professionals in higher education presented the Croatian Open Access Declaration. The Declaration emphasizes that OA to scientific information will increase visibility, influence, and prestige of Croatian science and culture (15).

## Institutional repository

Institutional repository (IR) is an online archive that captures and preserves works created by members of an institution, providing access to them in digital form (16,17). The Scholarly Publishing and Academic Resources Coalition (SPARC®) (18) defined IR as a digital archive of the entire intellectual output of members of a university, available to end-users inside or outside the university. The content of an IR has to be institutionally defined, scholarly, cumulative, perpetual, open, and interoperable (19). IR can host peer-reviewed articles published by scientific and professional journals, as well as other content, such as theses, professional and technical reports, conference proceedings, data sets, etc. By concentrating the intellectual products of an institution IR provides:

1) greater visibility of the scholarly work, increasing the impact and prestige of the authors and their institution in the scientific community,

2) support to the scientific and educational process in the institution,

3) public insight into the institutional intellectual output, also important to the funding bodies or sponsors, and

4) cost reduction in acquisition of scientific literature (20-23).

IR is an important part of the modern scientific and academic institutions’ digital infrastructure. The current list of the Directory of Open Access Repositories (Open Doar) includes 2525 institutional repositories (24), four them from Croatia: University of Zagreb Medical School Repository, FULIR – Full-text Institutional Repository of the Ruđer Bošković Institute, Repository of the Faculty of Mechanical Engineering and Naval Architecture, and Faculty of Humanities and Social Sciences Institutional Repository.

## University of Zagreb Medical School Repository

Building and maintaining an IR requires similar skills and abilities as building and maintaining a library collection, so it is not surprising that initiators and administrators in 80% of IRs are libraries (23).

In the early 2005, the Central Medical Library (CML) affiliated to the University of Zagreb School of Medicine, launched a project to design and develop an IR with the following main goals:

1) capturing institutional publication production, enhancing its accessibility, visibility, and distribution,

2) facilitating scientific communication between scientists and students in the field of medicine.

University of Zagreb Medical School Repository (25) fulfils the basic requirements listed in the SPARC ® position paper (19):

1) institutional affiliation,

2) scholarly content (full texts of theses, articles published in peer-reviewed journals, conference presentations, book chapters, etc),

3) cumulative nature and long-term preservation,

4) open access to all content,

5) interoperability supported by an open source software (EPrints) (26) and the OAI-PMH protocol (Open Archives Initiative Protocol for Metadata Harvesting).

Currently, the repository contains 1350 items, 963 of which are articles, 374 PhD theses, 1 book, 6 book chapters, and 6 conference communications, covering the period from 2003 onwards.

IRs are designed as networking platforms, where authors usually self-archive their works. However, authors are not prone to self-archiving, even when it is mandatory (27,28). Therefore, the CML developed an alternative strategy of document collection. By searching the available resources (bibliographic databases, catalogs, internal documentation, etc), librarians first identify the publications. After reviewing the publisher’s policy regarding OA, the librarian contacts the authors, seeking permission and an appropriate version of the publication that will be deposited to the repository. Special attention is paid to detailed compliance with copyright and licensing restrictions. This particularly applies to the version that can be archived (usually the final author's draft version, before or after peer-review), proper reference to copyright holders, and embargo imposed by certain publishers. By agreement with the editorial boards of the important Croatian medical journals, the CML archives the publishers’ version of all papers authored by the members of the University of Zagreb School of Medicine. The self-archiving option is blocked and archiving is done by librarians. This archiving method will be used until authors become aware of the importance of the IR for personal, institutional, and scientific promotion. The benefits of the IR research dissemination have so far been recognized and actively supported only by a few, mostly younger, teachers and researchers.

PhD theses comprise 28% of the repository's content, and it is the single access point to their full-text versions. The University of Zagreb has still not implemented the OA policy and postgraduate students are not required to archive their theses. Therefore, the CML has an important role in encouraging them to permit their depositing to IR. The CML strictly respects students' intellectual property rights. When submitting a thesis manuscript to the School’s administration the students fill out a permission form and decide whether they want to deposit their work immediately or one year after they obtain a doctor’s degree, or whether they refuse to give permission. From 2003 to 2013, 657 students obtained a PhD and 56.9% (374/657) granted their permission to deposit the thesis to IR.

The success of an IR depends on the authors’ willingness to archive their works. Authors’ lack of familiarity with the concept of open access and worrying about copyright issues may be a major barrier to IR development. Therefore, it is necessary to continually promote the purpose and advantages of archiving in a repository. In 2013, the University of Zagreb Medical School Repository was accessed 132 000 times (monthly access between 9000 and 13 000 times). The users mostly searched for PhD theses. These results are consistent with other studies (29). In contrast to journal articles, which “live” through multiple sources (journals, bibliographic databases), PhD theses are accessible primarily through IR (30).

## Conclusions

The University of Zagreb School of Medicine has been supporting OA since 1996, first through the *Croatian Medical Journal* and later through its IR (31). When the University of Zagreb Medical School Repository was included into DRIVER – the network of European repositories (now OpenAIRE), it became a part of a powerful and flexible infrastructure of pan-European digital repositories, available to researchers, administrators, and the general public (32). Such wider outreach is especially important for the developing and peripheral scientific communities, where the main publishing outlet are local publications. The University of Zagreb Medical School Repository ranked in the middle of the list of the Ranking Web of Repositories, 13th edition (743 of 1563), a tool measuring repositories’ visibility and echo (33). This implies that an IR can serve not only as a publication archive and dissemination platform, but also as a marketing tool for increasing institutional visibility and prestige.
